# Neural rhythmic symphony of human walking observation: *Upside-down* and *Uncoordinated* condition on cortical theta, alpha, beta and gamma oscillations

**DOI:** 10.3389/fnsys.2014.00169

**Published:** 2014-09-18

**Authors:** David Zarka, Carlos Cevallos, Mathieu Petieau, Thomas Hoellinger, Bernard Dan, Guy Cheron

**Affiliations:** ^1^Laboratory of Neurophysiology and Movement Biomechanics, Université Libre de BruxellesBrussels, Belgium; ^2^Department of Neurology, Hopital Universitaire des Enfants reine Fabiola, Université Libre de BruxellesBruxelles, Belgium; ^3^Laboratory of Electrophysiology, Université de Mons-HainautBruxelles, Belgium

**Keywords:** ERP, ERSP, ITC, SSVEP, walking, observation, virtual reality

## Abstract

Biological motion observation has been recognized to produce dynamic change in sensorimotor activation according to the observed kinematics. Physical plausibility of the spatial-kinematic relationship of human movement may play a major role in the top-down processing of human motion recognition. Here, we investigated the time course of scalp activation during observation of human gait in order to extract and use it on future integrated brain-computer interface using virtual reality (VR). We analyzed event related potentials (ERP), the event related spectral perturbation (ERSP) and the inter-trial coherence (ITC) from high-density EEG recording during video display onset (−200–600 ms) and the steady state visual evoked potentials (SSVEP) inside the video of human walking 3D-animation in three conditions: *Normal*; *Upside-down* (inverted images); and *Uncoordinated* (pseudo-randomly mixed images). We found that early visual evoked response P120 was decreased in *Upside-down* condition. The N170 and P300b amplitudes were decreased in *Uncoordinated* condition. In *Upside-down* and *Uncoordinated* conditions, we found decreased alpha power and theta phase-locking. As regards gamma oscillation, power was increased during the *Upside-down* animation and decreased during the *Uncoordinated* animation. An SSVEP-like response oscillating at about 10 Hz was also described showing that the oscillating pattern is enhanced 300 ms after the heel strike event only in the *Normal* but not in the *Upside-down* condition. Our results are consistent with most of previous point-light display studies, further supporting possible use of virtual reality for neurofeedback applications.

## Introduction

Neuronal processing of the visual system allows us to perceive objects, movements, colors, contrasts, and to represent the space around us with a very high resolution. In addition to the classical dichotomy between the ventral stream (the “What” pathway) supporting object vision and a dorsal stream (the “Where” pathway), a more recent conception based on clinical evidence (Kravitz et al., 2011) divides the dorsal stream into three sub-pathways projecting on to the premotor (supporting visually-guided actions), the prefrontal and the medial temporal lobes (supporting spatial working memory) both directly and through the posterior cingulate and retrosplenial areas (supporting navigation). This emphasizes the contribution of numerous functionally specialized, hierarchically organized visual areas giving rise to a conscious perception of the different attributes of the visual scene (Zeki et al., 1991; Singer, 1999). The discovery of the phase-locking mechanism at the level of the cortical neurons producing gamma oscillation (Gray et al., 1989) constitutes a strong scientific foundation for the binding by synchrony hypothesis (Singer, 1999) and has also paved the way for non-invasive investigation of the implicated mechanisms by electroencephalography (EEG) and event-related potentials (ERP) (Makeig et al., 2002; Cheron et al., 2007, 2014; Cebolla et al., 2009, 2014). New approaches of signal analysis (Delorme and Makeig, 2004) have permitted to better understand the genesis of the sensory evoked responses including visual motion in VR environment (Gramann et al., 2009; Cheron et al., 2014) and the origin of the movement gating of sensory evoked responses (Cebolla et al., 2009).

The discovery of mirror neurons responding similarly when the monkey performs an action and when it observes the experimenter performing the same action (Rizzolatti et al., 1996) has led to human studies of visual processes involved in recognition (Blake and Shiffrar, 2007; Avanzini et al., 2013; Di Dio et al., 2013), prediction of others' movements (Csibra, 2007; Kilner et al., 2007), and their implication in social cognition (Jacob and Jeannerod, 2005; Schütz-Bosbach and Prinz, 2007; Heyes, 2010; Press et al., 2011). Behavioral, neuroimaging and neurophysiological data have demonstrated a high sensitivity to reference frame (Pavlova and Sokolov, 2000; Pavlova et al., 2004; McGlothlin et al., 2012), human body form (Downing et al., 2001), kinematics of human movement (Avanzini et al., 2012; McAleer et al., 2014), gender and personal traits (Pollick et al., 2005; Troje et al., 2005; McGlothlin et al., 2012). Several studies demonstrated that shape and motion information are treated separately by ventral and dorsal visual streams, and converge to the posterior portion of superior temporal sulcus (Vaina et al., 2001; Giese and Poggio, 2003; Blake and Shiffrar, 2007). Moreover, the motor theory of perception, based on the fact that movement perception is influenced by the implicit knowledge about the working principles of the motor control system (Viviani and Stucchi, 1992; Rizzolatti and Craighero, 2004), give a critical place to ventral premotor cortex in biological motion perception processes (Saygin et al., 2004).

By extending Darwin's evolutionary perspective about face emotion (Darwin, 1872) to human locomotion, we may advance that the recognition of the human primate by its bipedal locomotion already present in early hominid before stone tools and large brains (Leakey and Walker, 1997) is probably one of the most vital activities of human in a selection retrospective view. Moreover, such human gestures may represent a constitutive element of the emotional body language (De Gelder, 2006, for a review). This whole body movement of *homo sapiens* is characterized by distinctive patterns of smooth, regular, alternated lower and upper limbs movements performed around a relatively fixed and erected posture of the head and trunk segment (Pozzo et al., 1990). Another remarkable element is the heel-strike considered as an acquired character of African and Asian apes linked closely to knuckle walking quadrupedalism (Thorpe et al., 2007; Crompton et al., 2010). These highly recognizable elements would implicate that human mirror neuron systems should be active when watching somebody else walk (Cheng et al., 2005).

Thanks to dynamics analysis of high-density EEG associating event-related potential (ERP), event-related spectral perturbation (ERSP) and inter-trial coherency (ITC), it has become possible to identify electrophysiological mechanisms related to recognition processes (Engel et al., 1997; Singer, 2009). In this context, coherent stimulus representation, including biological motion (Pavlova et al., 2004), are thought to result from binding of widely distributed cell ensembles by synchronizing their high-frequency oscillation activity (Tallon-Baudry and Bertrand, 1999; Singer, 2009). In parallel, other oscillatory processes may be activated following a motor template as suggested for mu rhythm (Ulloa and Pineda, 2007; Arnstein et al., 2011; Braadbaart et al., 2013; Urgen et al., 2013; Frenkel-Toledo et al., 2014). Point-light display of human locomotion has been used to characterized the cortical activity involved in recognition of locomotion either through MEG (Pavlova et al., 2004, 2006) or ERP studies (Hirai et al., 2003, 2005, 2013; Jokisch et al., 2005; Hirai and Hiraki, 2006; Krakowski et al., 2011; Buzzell et al., 2013).

Here, we studied the ERP and dynamics of theta, alpha, beta and gamma oscillations induced by the observation of an animated avatar in a virtual reality (VR). We hypothesized that physical plausibility of the spatial-kinematic of human locomotion plays a major role in different modes of neural processing (bottom-up and top-down) implicated in locomotion recognition: we expect that these processes will be reflected in different contributions of rhythmic power and phase-locked perturbation in different frequency bands and cortical areas. To address this question, we used an animation representing a human mannequin during walking action performed in normal, *Uncoordinated* kinematics and in *Upside-down* views. This will offer the possibility to extract the dynamic signature of human walking observation with respect to the neural activity evoked by the same image content but in an unusual frame of reference (*Upside-down* view) or without respect to normal kinematics (*Uncoordinated* walk) from the EEG signals.

## Materials and methods

### Articipants

Sixteen healthy volunteers took part in this study (ten males, mean age 25.9 years, range 18–35 years). All subjects were right-handed, had *Normal* or corrected-to-*Normal* vision, were naive with respect to the purpose of the experiment, and gave informed consent. The experiment was performed with the approval of the ethics committee of Université Libre de Bruxelles and realized in accordance with the ethical standards of the 1964 Declaration of Helsinki.

### Stimuli

**Figure 1:**
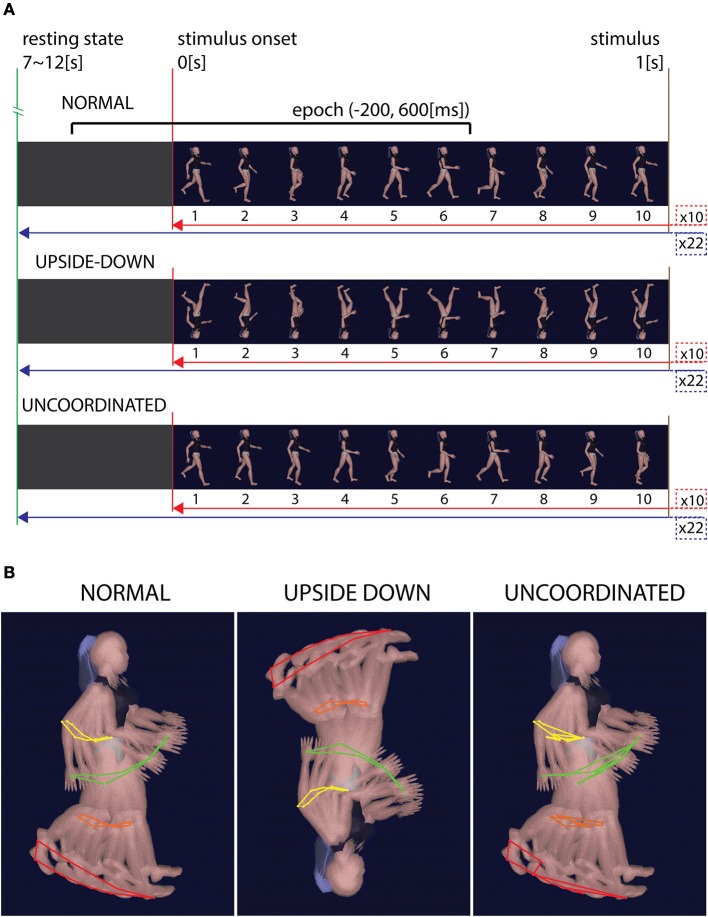
**Condition's stimuli. (A)** Time representation of the stimuli in each three conditions. *Normal* condition shows ten successive gait cycles of 1 s each. Trials were separated by 7–12 s of gray screen and repeated 22 times. *Upside-down* condition show the same images sequence, rotated to 180° in the image plane. *Uncoordinated* condition show the same first image as the *Normal* condition while all the others images were mixed. **(B)** Kinematics representation of the gait in the three conditions. While *Normal* and *Upside-down* conditions have the same coherent kinematic sequence, *Uncoordinated* condition show an incoherent kinematic sequence.

Visual stimuli consisted of an animation in the center of the screen representing human walking mannequin (from Cal3D Library) that was presented in three different ways: *Normal* walking (N), *Upside-down* (U), and *Uncoordinated* (J) (Figure 1). Each animation was organized in 22 blocks of 10 s duration interspersed by 7–12 s of random periods of gray screen. This allowed us to obtain a repetitive control of the baseline state throughout the whole recording session and facilitated the recording of the averaged responses (see below). For the *Normal* walking condition, each block was initiated by an image representing the heel strike event of the right leg and was ended by the last image preceding the next heels strike of the right leg picture (Figure 1A). The animation had a 10 Hz frequency. In the *Upside-down* condition the same successive pictures were merely inverted, keeping the same kinematics sequences. In the *Uncoordinated* condition the first image remained the same as the *Normal* condition while all the others were randomly mixed giving rise to an incoherent *Uncoordinated* movement. The random sequence was conserved for each trial and each subject (Figure 1B).

We have calculated the luminosity of each image in the three colors (RGB) by using “imread” MATLab function. This function stores in a matrix the composition in red, green and blue of each pixel of an image. For each frame of the animation, we subtracted this matrix to that of the previous frame. We have then computed the mean of the resulting matrix and called this composition “dynamic contrast.” This represents overall change between two successive frames of the composition in luminosity of the same located pixels.

### Experimental setup

The recording was realized in a single session. EEG was recorded in 128 channels (ANT system, *The Netherlands*) at a sampling frequency of 2048 Hz and with a resolution of 22 bits (71.5 nV per bit). An active-shield cap using 128 Ag/AgCl sintered ring electrodes and shielded co-axial cables (5–10 electrode system placements) was comfortably adjusted to subject head. All electrodes were referred to left earlobe. Impedance was lowered below 10 kΩ for each electrode and checked before each recording. Displays were presented on a 17” Dell computer screen. Participants looked straight ahead at the computer screen through a form-fitting facemask and a circular barrel (cylinder). The screen was centered on the line of gaze at a distance of ~30 cm from the eyes. Viewing through the barrel removed any external visual references. In addition, subjects had earplugs to isolate from external hearing disturbance. For eight subjects, we presented in three successive sessions the *Normal* walking condition, then *Upside-down* walking and then *Uncoordinated* one *(No-Random group)*. For eight other subjects, conditions were presented randomly in three successive sessions (*Random* group). We made a pause between each session in order to limit the effect of fatigue. As the aim of this study was to evaluate the effect of purposeless perception on the brain rhythms, no particular attentional task was required. However, the state of awareness was continuously checked by online EEG (absence of slow rhythm linked to drowsiness) and EOG, for which we placed electrodes above, below, right and left of the right eye. In particular, we checked that the blink number and configuration remained unchanged throughout the experimental session. We used an in-house script that counts the number of blinks by incrementing an index for each potential higher than 250 μV, and calculates the interval between blinks to provide a view of their configuration over time. We then calculated the number of saccades by EOG derived function. The results show there is no difference between conditions for blink (means by subject for *Normal*: 81 ± 33.3; *Upside-down*: 99.8 ± 27.2; *Uncoordinated*: 120.4 ± 34.6) and saccades (means by subject for *Normal*: 316 ± 137.8; *Upside-down*: 455.4 ± 98.3; *Uncoordinated*: 385.2 ± 136.6).

### Data treatment

Off-line data treatment and analysis was performed by means of EEGLAB software (Delorme and Makeig, 2004; Brunner et al., 2013) and in-house MATLAB-based tools (Cheron et al., 2014). DC offset was removed, then band pass filter 0.1–80 Hz and notch filter around 50 Hz (47.5–52.5 Hz) were applied to attenuate electrical artifacts. Portions of data and defective electrodes (max. 6%) were removed by careful visual inspection. Ocular (blink and saccade) and any other remaining artifacts (muscular, cardiac) were isolated by ICA algorithm decomposition. We used the scalp topography, temporal activity localization and spectra magnitude criterion to identify ICA related to artifact. In case of doubt the rejection occurred only if all experimenters involved in data treatment reached agreement. After ICA rejection, defective electrodes were spherically interpolated.

Two analyses were performed: animation onset analysis and SSVEP analysis. In the animation onset analysis, data were organized in epochs corresponding to intervals [−1000; 3000] ms, centered on animation onset. We rejected epochs according to ±100 μV threshold criterion, and we made a visual review to confirm epoch rejection. In total, we obtained 17 ± 5 epochs per subject (*n* = 16) and per condition (*n* = 3). A study design was used to average data from subjects for each condition. A time window of 1000 ms before stimulus onset was used as baseline.

In SSVEP analysis, data were organized in epochs corresponding to intervals [−200; 600] ms, centered on each heel strike except for the first and the last one. As in preceding analysis, we applied ±100 μV threshold criterion confirmed by a visual review. In total, we obtained 159 ± 16) epochs per subject (*n* = 16) and per condition (*n* = 3). We performed a grand average study including 2065 ± 26 trials for each condition. The interval [−200; 0] ms was used as baseline, and the first and last heel strikes were excluded from the analysis In this case, the SSVEP analysis was independent of the neutral black screen periods allowing to join mixed *Random* and *Non-Random* groups.

A subset of 32 electrodes was explored for each measure analysis: O2, Oz, O1, POz, P8, P4, Pz, P3, P7, CP6, CP2, CP1, CP5, T8, C4, Cz, C3, T7, FC6, FC2, FC1, FC5, F8, F4, Fz, F3, F7, Fp2, Fpz, Fp1. We first checked ERP and ERSP of [−1000; 3000] ms epoch, and then we focused on events related to animation onset, and SSVEP centered on heel strike between −200 and 600 ms. ERP, ERSP, and ITC analysis was performed. Difference between *Random* and *No-Random* groups and between *Normal* and both *Upside-down* and *Uncoordinated* condition were performed by EEGLab non-parametric permutation test (*n* = 2000) at each trial latency of the average ERPs and every time-frequency point for ERSP and ITC.

## Results

### Event-related potential

**Figure 2:**
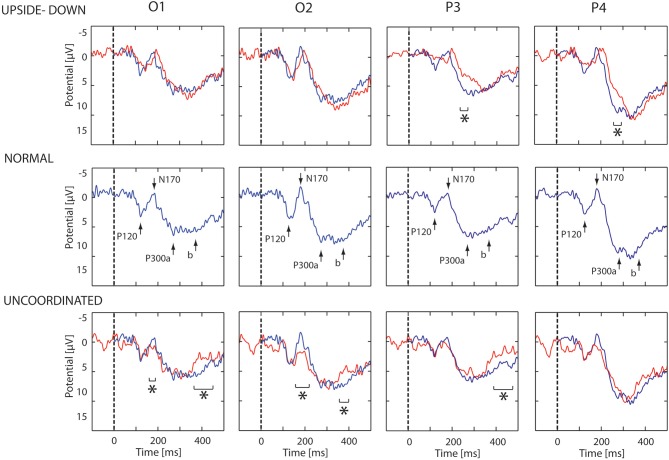
**ERP evoked by animation onset in each condition**. ERP recorded in O1, O2, P3, and P4 electrodes. In middle part, the ERP evoked by the normal animation onset show three successive potentials: P120, N170, and P300a,b. In upper part, the blue and red lines show respectively the ERP response in the *Normal* and *Upside-down* condition. In lower part, the blue and red lines show respectively the ERP response in the *Normal* and *Uncoordinated* conditions. Significant differences are mark by asterisks (*p* < 0.05).

The first noticeable ERP component referenced to the earlobe elicited after the onset of the VR-animation was the P120 component recorded in occipito-parietal electrodes. Analysis revealed a peak reduction in *Upside-down* condition with respect to *Normal* condition over occipito-parietal regions. The next component was the N170 mainly recorded over the occipito-parietal regions (Figure 2). We observed a significant decrease in *Uncoordinated* condition over occipital and parieto-lateral regions. This was followed by a large P300 component ending at about 500 ms in occipital regions and 400 ms in frontal regions. In order to simplify the description of condition effect, we subdivided P300 in the two classical P300a and P300b components (Figure 2). The P300a component was significantly smaller in the *Upside-down* condition over parietal regions with respect to the *Normal* situation, whereas the P300b component remained the same. On the contrary in the *Uncoordinated* condition, the P300a component was not modified whereas the P300b showed a significant decrease in occipital and parietal regions (Table 1). Comparison between *Random* and *No-Random* group revealed no significant difference between their respective conditions except in the P300 amplitude in parietal region which was greater for the *No-Random* group for the 3 conditions.

**Table 1:**
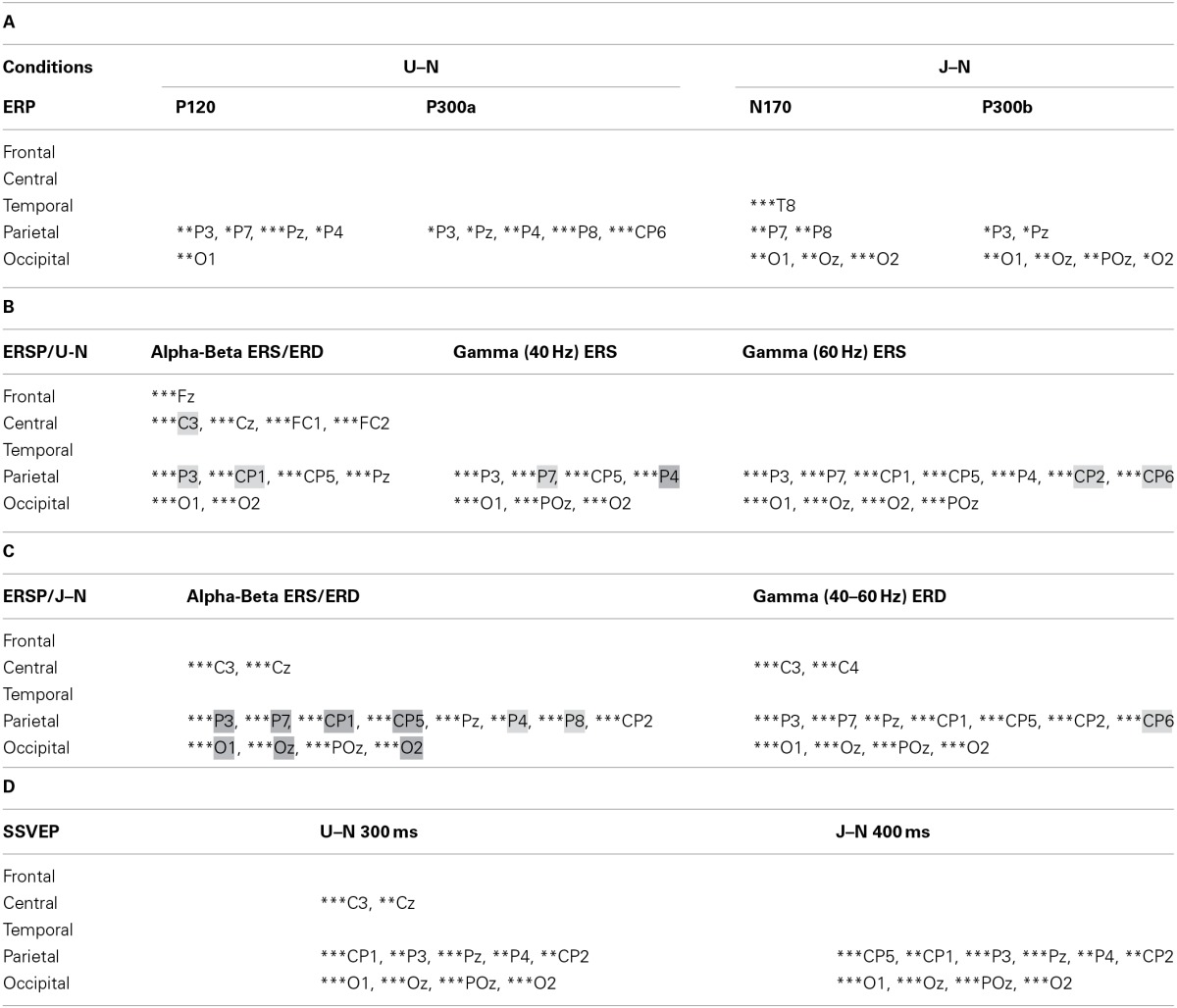
**Summary of statistical analysis about the 32 selected electrodes for both conditions *Upside-down* (U) and *Uncoordinated* (J) vs. Normal (N)**.

(A) ERP for P120, N170, P300a, and P300b amplitudes. (B) ERSP and ITC for U vs. N. (C) ERSP and ITC for J vs. N. Only the electrodes showing significant results are represented (^***^0.001, ^**^0.01). The reduction in theta phase locking (ITC) is indexed by gray shading (dark p < 0.001, light p < 0.01). (D) SSVEP behavior differences.

### Event-related spectral perturbation and inter-trial coherence

**Figure 3:**
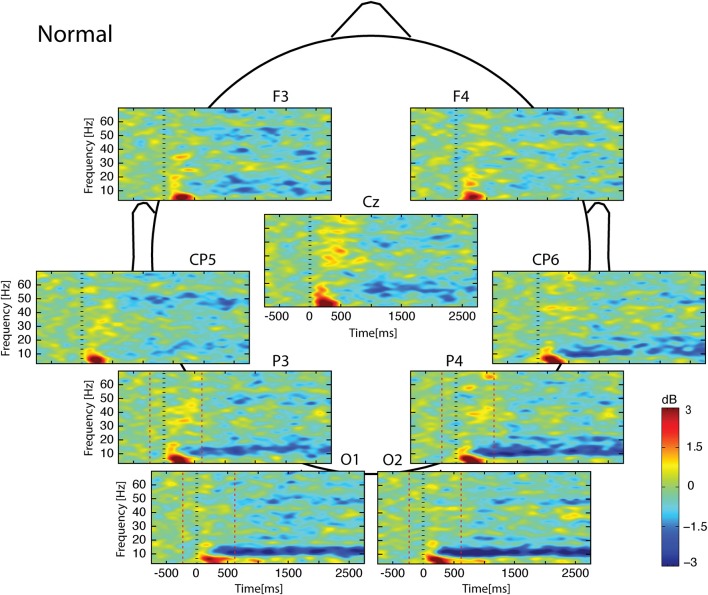
**Scalp ERSP on [−1000; 3000] ms in *Normal* condition**. Rhythmic activity can be splits in two parts: [0; 500] ms which contains responses evoked by animation onset; and [500; 3000] ms of maintaining task. Note that bilateral alpha-beta ERD was continue from 200 to 3000 ms, and gamma ERD at about 700 ms, maintained for the 3000 ms in the left sensori-motor region. Red lines delimit epochs analyzed: [−200; 600] ms.

**Figure 4:**
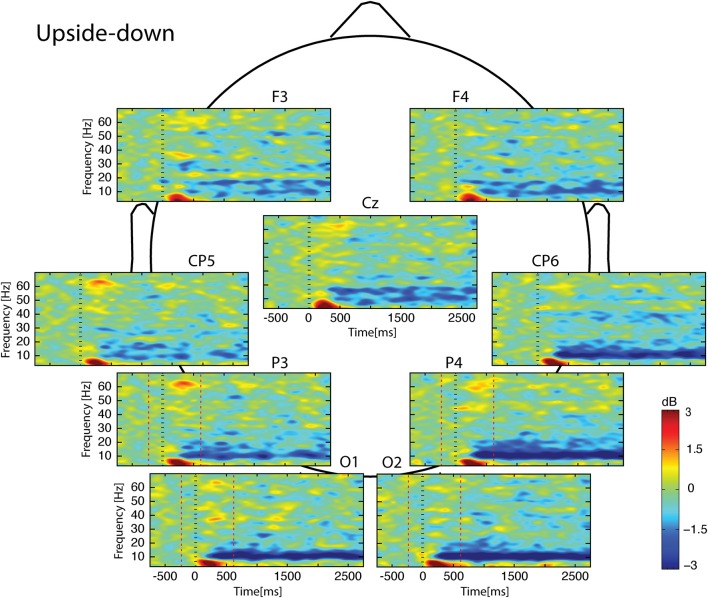
**Scalp ERSP on [−1000; 3000] ms in *Upside-down* condition**. Rhythmic activity can be splits in two parts: [0; 500] ms which contains responses evoked by animation onset; and [500; 3000] ms of maintaining task. Note that bilateral alpha-beta ERD was continue from 200 to 3000 ms. Red lines delimit epochs analyzed: [−200; 600] ms.

**Figure 5:**
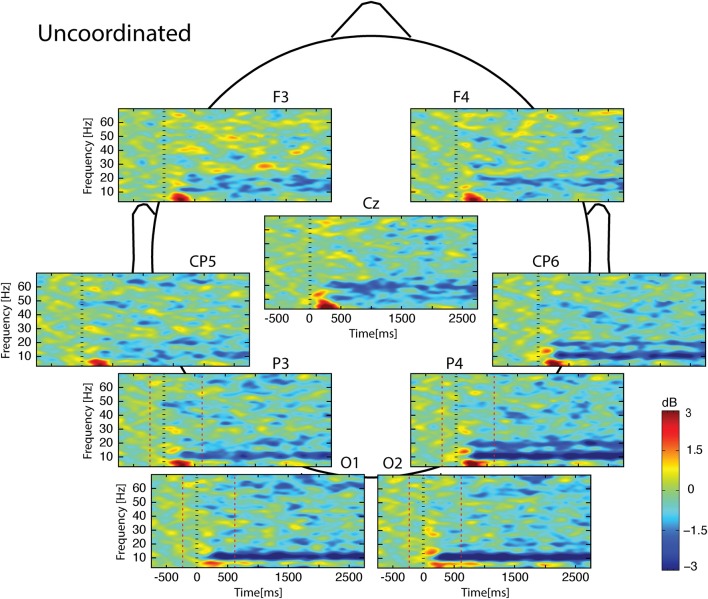
**Scalp ERSP on [−1000; 3000] ms in *Uncoordinated* condition**. Rhythmic activity can be splits in two parts: [0; 500] ms which contains responses evoked by animation onset; and [500; 3000] ms of maintaining task. Note bilateral that alpha-beta ERD was continue from 200 to 3000 ms. Red lines delimit epochs analyzed: [−200; 600] ms.

Whatever the observed condition (Figures 3–**5**), the video onset triggered throughout the scalp a theta ERS in the [0; 500] ms interval followed by an alpha ERD initiated at about 200 ms and maintained during all the duration of the video. This alpha ERD was accompanied by a beta ERD which was more pronounced in the *Uncoordinated* condition (**Figure 5**). The *Normal* presentation induced a gamma ERD at about 700 ms and maintained for all the duration of the video in the left sensori-motor region (Figure 3, CP5 electrode). This gamma ERD was not present in the two others conditions (Figures 4, 5).

**Figure 6:**
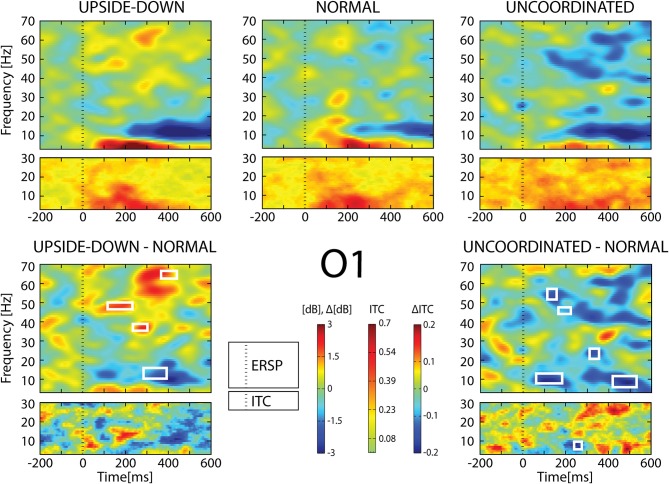
**ERSP and ITC from O1 electrode**. The upper panel shows the ERSP and ITC in each three conditions. The lower panel shows difference *Upside-down-Normal* (left), and *Uncoordinated-Normal* (right) for ERSP and ITC. Areas of statistical significances (*p* < 0.001) are marked by white squares.

**Figure 7:**
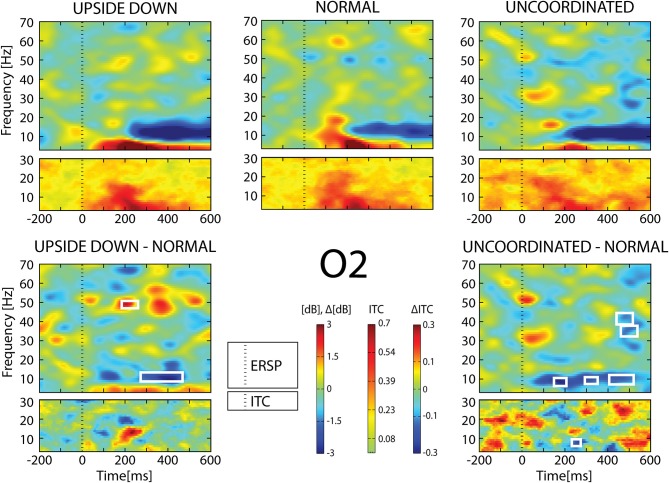
**ERSP and ITC from O_2_ electrode**. The upper panel shows the ERSP and ITC in each three conditions. The lower panel shows difference *Upside-down-Normal* (left), and *Uncoordinated-Normal* (right) for ERSP and ITC. Areas of statistical significances (*p* < 0.001) are marked by white squares.

**Figure 8:**
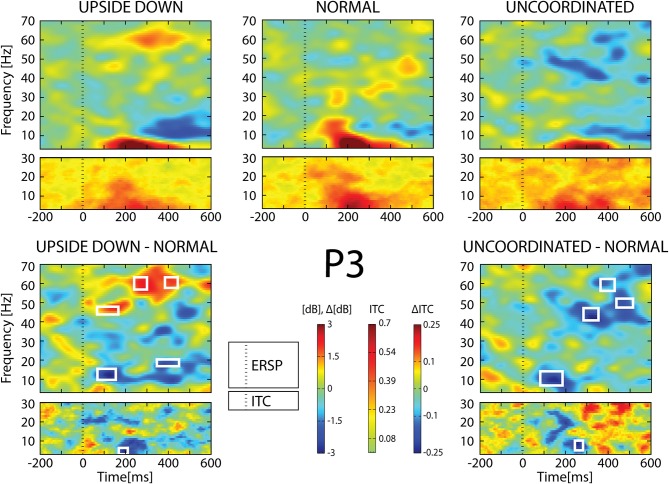
**ERSP and ITC from P_3_ electrode**. The upper panel shows the ERSP and ITC in each three conditions. The lower panel shows difference *Upside*-*down*-*Normal* (left), and *Uncoordinated*-*Normal* (right) for ERSP and ITC. Areas of statistical significances (*p* < 0.001) are marked by white squares.

**Figure 9:**
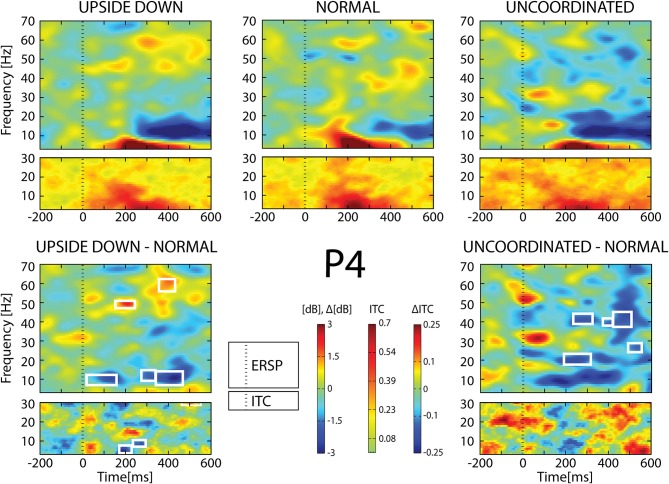
**ERSP and ITC from P_4_ electrode**. The upper panel shows the ERSP and ITC in each three conditions. The lower panel shows difference *Upside*-*down*-*Normal* (left), and *Uncoordinated*-*Normal* (right) for ERSP and ITC. Areas of statistical significances (*p* < 0.001) are marked by white squares.

In the [−200;600] ms time-window, the *Normal* condition was characterized by the following observations (Figures 6–9): (1) at the latency of the P120, ERSP plots showed an earlier alpha ERS reaching maximal value in the parietal region and extending to the beta band in the parieto-central region. (2) This was sustained by theta activation and phase-locking presenting its maximal value at about 200 ms. (3) ERD in the upper alpha band at about 200 ms in the occipito-parietal regions. (4) ERS clusters in the gamma range (30–70 Hz) in the parieto-occipital and centro-frontal regions.

With respect to the *Normal* condition, in the *Upside-down* condition, three significant changes were noted (Figures 6–9): (1) a decrease in alpha-beta power between 100 and 500 ms over occipital to frontal regions, resulting in a lack of ERS in alpha bands at the latency of the P120, and an increased alpha-beta ERD at about 200–500 ms; (2) a decrease of the theta phase-locking in the parieto-central regions; (3) an increase in (gamma) 40 and 60 Hz ERS over occipito-central regions, respectively at about 150 and 350 ms latency (Table 1).

With respect to the *Normal* condition, in the *Uncoordinated* condition the following was observed (Figures 6–9): (1) a decrease of alpha-beta band at the latency of the P120, followed by an earlier and greater alpha-beta ERD over the occipito-parietal region; (2) a reduction of theta phase-locking. However, in contrast to the *Upside-down* and *Normal* condition, (3) the *Uncoordinated* animation produced a gamma 40–60 Hz ERD at about 200–500 ms over occipito-central regions (Table 1). The analysis of the dynamic contrast of the image showed that the *Uncoordinated* condition presented an increased contrast between the third and the fourth image (400 ms after the onset) with respect to the two other conditions.

### Steady state visual evoked potentials

**Figure 10:**
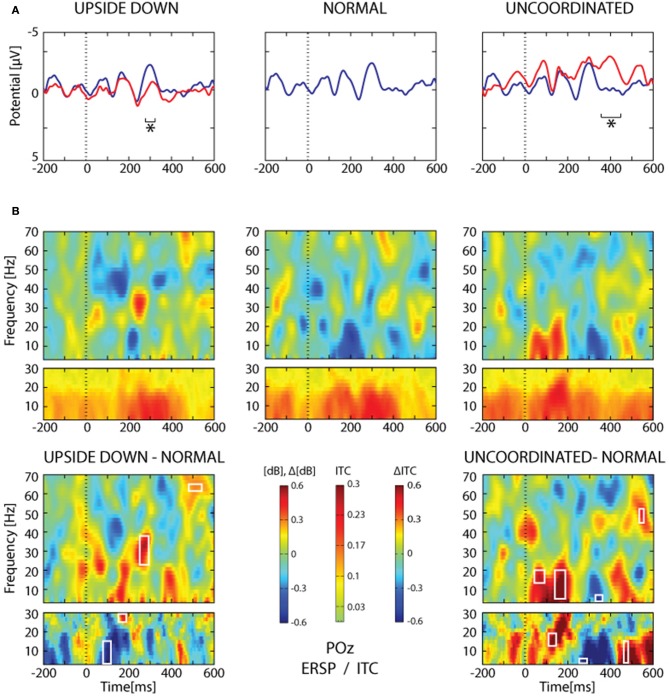
**SSVEP analysis from POz electrode. (A)** Grand average trace corresponding to 16 subjects and about 2065 ± 26 trials for each condition. The heel strike of the right leg was used as trigger. Note the oscillating pattern at about 9 Hz and the superimposition of the *Normal* condition (blue trace) over the *Upside-down* (red trace, left part) and the *Uncoordinated* condition (red trace, right part). Areas of statistical significance (*p* < 0.001) are indicated by an asterisk. **(B)** The upper panel shows ERSP and ITC analysis of the same SSVEP data for the three respective conditions presented in **(A)**. The lower panel shows, ERSP and ITC differences *Upside-down*-*Normal* (left), and *Uncoordinated-Normal* (right). Areas of statistical significance (*p* < 0.001) are marked by white squares.

When the heel strike of the right leg was used as trigger, the average trace corresponded to an oscillatory pattern peaking at about 9 Hz. (8.86 Hz). This was observed for all subjects and conditions and may be considered as a SSVEP induced by the frequency of the video. Figure 10 illustrates the SSVEP traces resulting from a grand average of all the 16 subjects which conserve the 9 Hz oscillating pattern presented in each single subject. However, the amplitude of the grand average oscillation was not constant throughout the time period. The first negative peak occurred close to 100 ms after the heel strike in any of the 3 different conditions (Figure 10A). For *Normal* and *Upside-Down* condition the SSVEP amplitude increased after the heel strike and culminated at a latency of 300 ms (negative peak) only in the *Normal* condition. Thereafter, the oscillating pattern decreased in *Normal* and *Upside-down* condition but was maintained in the *Uncoordinated* condition.

The *Upside-down* condition showed a significant decrease of the third negative SSVEP component at 300 ms with respect to the *Normal* condition over parieto-occipital regions (Figure 10A, *left part*; Table 1). The reduction of the ascending phase slope of this negativity in the *Upside-Down* condition was accompanied over parieto-occipital regions by a power increase in beta-gamma band at about 250 ms and later by a power increase in gamma band (60 Hz) at about 400 ms (Figure 10B, *left part*). These effects were preceded by a decrease of theta-alpha bands ITC at about 120 ms with respect to the *Normal* condition (Figure 10B, *left part*).

The *Uncoordinated* condition showed a significant increase of the SSVEP negativity at about 400 ms with respect to the *Normal* condition (Figure 10A*, right part*; Table 1). This was preceded by an ERD in the theta–alpha band (Figure 10B) over parieto-occipital regions. While the higher amplitude of the first negative peak recorded in this condition remained under the significance level (Figure 10A, *right part*) it was accompanied by a significant increase of theta-alpha bands power at about 100 ms with respect to the *Normal* condition. These effects were followed by successive decrease and then increase of theta-alpha ITC respectively at about 350 and 450 ms (Figure 10B, *right part*).

## Discussion

To our knowledge, this work represents the first study on the dynamic neural response elicited by VR-animation of human walking. The presentation of the VR-animation elicits ERP components classically described in response to a visual stimulation (Jeffreys, 1996). We demonstrate significant changes in the amplitude of the P120 and P300a when the avatar was upside-down, and the N170 and P300b when the walking sequence was perturbed (*Uncoordinated*) with respect to the *Normal* condition.

The presence of the early alpha ERS characterize the *Normal* condition. The alpha-beta ERD was reinforced and the theta phase-locking was disturbed in *Upside-down* and *Uncoordinated* condition. As regards gamma oscillation, a contrasting situation was seen as its power was increased in association with the *Upside-down* animation and decreased with the *Uncoordinated* animation.

An SSVEP-like response oscillating at about 9 Hz was also described when the heel strike event was used as trigger, showing that the oscillating pattern is enhanced 300 ms after the heel strike event only in the *Normal* but not in the *Upside-Down* condition.

### ERP

The present ERP components evoked by avatar observation are in accordance with those recorded in previous studies using point-light paradigm (Johansson, 1973). The timing of P120, N170, and P300 are commonly regarded as corresponding to the main three components P1 around 130 ms, N1 at 200 ms and N2 (P3 depending of the type and the placement of reference electrode) at 300–400 ms described in point-light walking studies (Hirai et al., 2003, 2005, 2009, 2013; Jokisch et al., 2005; Krakowski et al., 2011; Buzzell et al., 2013).

Although point-light and VR displays concern the same biological motion, VR display induced a stronger visual representation, including form and color than point-light walking. VR display conserved body structure in the *Normal, Upside-down*, and *Uncoordinated* conditions. This was not the case in point-light studies, where inverted condition associate form recognition to motion, and scramble condition deconstruct body form. In this context, the present VR-paradigm offers the possibility to dissociate recognition of body form occurring at the first video frame, and motion coherence (smoothness, coordination, speed, etc.) produced by the successive frames. This allows to focus specifically on the effect induced by changing the frame of reference in *Upside-down* and the global coherency of the walking motion in *Uncoordinated*.

The similarity of P120 evoked by the *Normal* and the *Uncoordinated* conditions is consistent with the fact that the first image is the same in these conditions. In contrast, point-light studies showed a delay and a decrease in P1 response elicited by scramble condition with respect to upright condition (Hirai et al., 2009, 2013; Krakowski et al., 2011). These results support common interpretation that first component reflects a global representation coding of form (Baccus et al., 2009; Buzzell et al., 2013; White et al., 2014). However, the finding of decrease P120 in the *Upside-down* condition with respect to the *Normal* condition was not reported by previous point-light studies. The origin of this early alteration can be explained by both bottom-up and/or top-down process. Distinctions between these influences are not easy. The *Normal* presentation of the avatar may unconsciously induce an easy visual representation than the *Upside-down* mannequin which implies a mental transformation of the reference frame. In parallel, the repeated presentation of normal or inverted locomotion can predictively influence this early response by a top-down effect exerted by the frontal cortex to the primary visual cortex (Peyrin et al., 2010; Cardin et al., 2011; Zanto et al., 2011; Ramalingam et al., 2013). However, previous studies suggest that explicitly attended tasks process does not appear to influence the earlier activity at about 100 ms (Krakowski et al., 2011; Buzzell et al., 2013).

Concerning later activation, N1 and N2 were generally related to integration of form and motion (Baccus et al., 2009; Buzzell et al., 2013; White et al., 2014). It is interesting to note that the effects of *Uncoordinated* condition on N170 (analogous to N1 in point-light studies) and of point-light scramble on N1 are comparable, as N170 was reduced in *Uncoordinated* condition as N1 in scramble condition (Hirai et al., 2003, 2013; Jokisch et al., 2005). This result suggests that N170 was mainly related to motion. In contrast, P300a (analogous to N2 in point-light studies) amplitude was the same in *Normal* and *Uncoordinated* conditions, whereas N2 was larger in upright point-light walker than point-light scramble (Jokisch et al., 2005; Hirai et al., 2013). Moreover, P300a amplitude was smaller in *Upside-down* than in *Normal*. This could also be related to the alteration of the SSVEP pattern occurring at this latency in the *Upside-down* vs. *Normal* condition. Taken together these results suggest that P300a was mainly related to the global form of walking.

Finally, the effect we recorded on P300b and in the SSVEP at about the same latency in the *Uncoordinated* condition is comparable to the late phase describe by Krakowski et al. (2011), which was characterized by a greater positivity in response to upright and inverted point-light walker than point-light scramble. This last phase after 400 ms is generally considered as indexing a high-order representation coding (Krakowski et al., 2011). This effect observed in *Uncoordinated* suggests that P300b component is sensitive to coherence of motion rather to mere recognition of walking.

### ERSP and ITC

#### Alpha ERS/ERD

The significant alpha ERS occurring at about 120 ms in the occipito-parietal regions characterized the *Normal* walking observation. It was followed by an alpha-beta ERD at 200 ms, which extends throughout the video. This was significantly more pronounced in *Upside-down* and *Uncoordinated* conditions than in *Normal*.

The first alpha ERS is in accordance with recent studies showing similar transient alpha increase in response to upright facial motion (Girges et al., 2014). The suppression of the early alpha ERS in *Upside-down* suggest that the inversion of the body presentation rapidly affect the early visual process. However, similar alteration found for the *Uncoordinated* condition while the first image was exactly the same suggests a top-down influence. Alpha oscillation has been interpreted as reflecting global inhibition of the cortex, improving behavioral performance by facilitation of the cognitive control (Klimesch et al., 1996, 2003, 2007; Klimesch, 1999, 2012; Cheron et al., 2006; Haegens et al., 2010). Thus, increase in alpha power (ERS) may participate to a general clearance of noise or distracting event in order to selectively update relevant incoming information (Sadaghiani et al., 2012), and access to memory (Klimesch, 2012). In this context, suppression of alpha ERS in *Upside-down* and *Uncoordinated* conditions would be correlated with increase of attention to motion cue and involvement of cognitive resources.

The next alpha ERD are in line with previous research showing decrease in alpha band power during perception of human motion (Cochin et al., 1998; Ulloa and Pineda, 2007). This was generally related to the desynchronization of mirror neurons activity as studied with EEG and fMRI combination (Arnstein et al., 2011), and would reflect a release from inhibition. However, it is interesting to note that our results are in contrast to studies of face perception (Girges et al., 2014), which report a greater alpha ERD in response to upright facial motion than in inverted condition. This difference between face and body motion recognition may be explain by high specialization of the brain to face recognition, and in particular to treatment of semantic content of facial gesture (Rojas et al., 2011). According to Klimesch et al. (1997, 1999, 2012), the alpha ERD increased as a function of the semantic content of retrieved information from the storage system. In our study, each stimulus has the same semantic content as a walking avatar. In this context, amplification of alpha ERD would indicate a recruitment of the mirror neurons system in order to recognize or predicted observed motion, by transformation of reference frame (in *Upside-down*) and reconstruction of motion (in *Uncoordinated*). The enhancement of the alpha ERD in *Upside-down* and *Uncoordinated* condition might then facilitate a dynamical process throughout the neural network involved in alpha rhythm generation evoked by the *Normal* walking avatar.

#### Theta ERS

The present ITC analysis shows that phase locking occur mainly in the theta range (peaked at ~5 Hz). However, as it is classically the case, this is not a pure phase locking because it was accompanied by theta ERS throughout all electrodes. Indeed, the visual evoked potentials (P100-N200) elicited by the classical checkerboard pattern or by more complex visual stimuli were accompanied by a clear theta ERS and related ITC (Klimesch et al., 2004; Cheron et al., 2014). Although present in each of the three present conditions in the 100–400 ms time period, the theta ITC was significantly perturbed in both *Upside-down* and *Uncoordinated* condition, while theta ERS were not significantly different. This indicates that the recognition of *Normal* walking is accompanied by a stronger theta phase locking peaking between 200 and 300 ms.

In humans the theta EEG rhythm (4–7 Hz) was initially defined as an intermediate rhythm between delta and alpha (Walter and Dovey, 1944; Mitchell et al., 2008). Later, the term FM-theta (FM for fronto-midline) was introduced by Ishihara and Yoshi (1972) when EEG was recorded during arithmetic task (Ishihara and Yoshi, 1972). Later, the presence of FM-theta during arithmetic and musical activities was demonstrated with MEG (Sasaki et al., 1996a,b,c). The midline frontal areas, such as the anterior cingulate cortex encompassing the lateral part of the prefrontal cortex are commonly cited as potential generators of the FM-theta (Gevins et al., 1997; Mizuhara et al., 2004; Sauseng et al., 2007).

In rat hippocampal regions, theta oscillation (3–9 Hz) is recognized to play an important role in the phase precession of the place cells firing assuming cued recall of the coming positions along the locomotion path of the rat (O'Keefe and Recce, 1993). The intrinsic theta generator of the hippocampal cortex is reinforced by the extrinsic theta pacemaker situated in the medial septal nucleus and allows a large-scale synchronization of theta oscillations in the hippocampus (Kocsis et al., 1999; Buzsáki, 2002). Theta oscillation is not restricted to the hippocampus but also emerges in different cortical areas in the rat (Leung and Borst, 1987; Silva et al., 1991). The ability of different cortical regions to produce theta is supported by slice recording demonstrating that theta oscillation may be produced by the activation of the NMDA receptors of the layer 5 (Silva et al., 1991; Flint and Connors, 1996) as well as by cholinergic activation of interneurons (Blatow et al., 2003).

Although human theta rhythm is not as robust as in the rat hippocampus, the ability of the human cortex to produce theta oscillation is now well recognized. It has been related to sensorimotor integration (Caplan et al., 2001), navigation (Kahana et al., 1999), memory load (Howard et al., 2003) and working memory (Raghavachari et al., 2001, 2006; Liebe et al., 2012). Interestingly, all the different phases of virtual movement during a navigation game induced an increase of 4–8 Hz oscillation in both the hippocampus and neocortex in human (Ekstrom et al., 2005). Although the present experiment involves the observation of human locomotion it cannot be assumed that the recorded theta oscillations are specifically related to locomotion *per se*. Indeed, theta oscillations are now considered as a basic physiological element involved in global oscillatory synchronization processes linking together multiple brain regions (Buzsáki and Draguhn, 2004; Fries, 2005). For example, the multiplicity of functional roles for this oscillation was demonstrated by the fact that the amplitude of theta power recorded over the temporal and frontal cortex predicted the behavioral performance of the subject (Sederberg et al., 2003). A recent MEG study demonstrated that hippocampal-prefrontal theta synchronization plays a mnemonic guidance in human decision-making (Guitart-Masip et al., 2013). Single neurons and local field potential recordings in the human medial temporal lobe show that theta phase locking reflects a global activation providing a temporal window for the conscious recognition (Rey et al., 2014). At a lower hierarchical level closer to the present observational task, theta oscillation is related to the perception of color shape of object and visual attention (Fries et al., 2001b). It is also involved in different sensory modalities to provide meaningful chunks of neuronal signals allowing subsequent decoding for an enhanced perception. In our case, such theta oscillation may thus be viewed as taking part of time-division multiplexing mechanism representing sequential information upon which a neuronal code may emerge by cross-frequency interaction with faster (gamma) oscillation (Akam and Kullmann, 2014).

#### Gamma modulation

Gamma oscillation (30–100 Hz) occupies a privileged position in cognitive neuroscience. The current understanding of gamma oscillation points to its emergence from the synchronous activity of a large ensemble of firing neurons (Eckhorn et al., 1988; Gray et al., 1989; Jensen and Colgin, 2007). It is central to the binding theory, in which gamma oscillations combine different features in a visual scene to form a coherent percept (Singer, 1999). Unexpectedly, our results show that *Upside-down* condition elicited gamma power increase at about 150 and 400 ms and a gamma ERD at the same latency in the *Uncoordinated* condition. This contrasting behavior of gamma oscillation is interesting because these oscillations are considered to underlie perception of coherent stimuli. These data are in accordance to previous MEG study showing enhancements in gamma rhythm at 100 ms after display onset in upright and inverted point-light walker (Pavlova et al., 2004). However, in the latter study additional gamma peak appeared only for upright point-light walker at 130 and 170 ms. Another study of the same group reported increased gamma activity in the left parieto-occipital region at 80 ms, with additional peaks in attended point-light walker on the right parietal and temporal cortex at 120 and 155 ms, respectively (Pavlova et al., 2006). The present results are in agreement with the data of Tallon-Baudry et al. (1996), where they demonstrated the presence of non-phase-locked gamma activation (60 Hz) at about 300–400 ms after the presentation of an illusory Kanizsa triangle figure (Tallon-Baudry et al., 1996). In this experiment, the gamma activity was stronger when the recognition task required additional mental reconstruction (stronger gamma oscillation for illusory triangle than normal triangle). In our present study, a non-phase-locked 60 Hz power increase occurred at the same latency only when the walking avatar was presented in *Upside-down* configuration. The subject was not instructed to perform any mental task but implicit recognition can recruit gamma activity for unconscious and conscious neuronal process (Aru et al., 2012; Vidal et al., 2014). The complex interplay between these neuronal qualia occupies a central position in cognitive neuroscience (Kandel, 2013). In the context of the Global Workspace Theory, serial and parallel processing take part from the widespread treatment of unconscious information to the emergence of consciousness (Baars, 1997; Dehaene and Naccache, 2001; Baars et al., 2013; Dehaene et al., 2014).

From a physiological perspective, experiments and modeling have demonstrated that gamma rhythms emerge from the interaction between local excitation and inhibition (Traub et al., 1997; Brunel and Wang, 2003; Kang et al., 2010), in which the gap junctions between interneurons play a pivotal role in ensuring gamma oscillation coherence (Traub et al., 2003; Whittington and Traub, 2003). In macaque, high density electrocorticography recording (Brunet et al., 2013) demonstrated that natural viewing induced a strong gamma oscillation (50–80 Hz) over most of the recorded visual cortex including V1 and V4 but not over most of the remaining cortex extending from superior temporal sulcus to the anterior part of the arcuate sulcus. The functional link between neuronal spikes and local field potential oscillation has been well documented in different preparations, demonstrating that spike-field coherence in the gamma-band frequency is accompanied by power enhancement of the gamma rhythm (Fries et al., 2001a,b, 2002, 2008). It was also demonstrated that when visual stimuli are moving smoothly, the visual cortex produces neuronal synchronization in the gamma-frequency band (Friedman-Hill et al., 2000). This gamma synchronization is considered as a key element for signal transmission to postsynaptic targets and to assume the continuity of the visual message (Fries, 2009).

In this context, the gamma ERD recorded during the *Uncoordinated* condition could be explained by a previous experience of Kruse and Eckhorn (1996) realized in the primary visual cortex of the cat. When a smooth movement of the visual field was presented it induced gamma oscillation, but when the smooth movement was intermingled with sudden random acceleration in and against the original direction of the smooth movement the gamma oscillations disappeared (Kruse and Eckhorn, 1996). This latter situation corresponds to the present *Uncoordinated* condition where gamma ERD replace gamma ERS present in *Normal* and *Upside-down* condition. The smoothness aspect of the walking video for both *Normal* and *Upside-down* presentations induces gamma oscillation while the sudden “*Uncoordinated*” image desynchronizes the neuronal population responsible for the gamma oscillation. In addition, Kruse and Eckhorn (1996) have demonstrated an inverse relationship between the decrease in gamma power and an increase in the stimulus-locked responses in lower frequency band (Kruse and Eckhorn, 1996).

Following the canonical microcircuit model (Bastos et al., 2012) based on intracellular recordings in cat visual cortex incorporating the neuronal sources of forward and backward connections in cortical hierarchies, it was proposed that the superficial pyramidal neurons generate gamma responses whereas deep pyramidal neurons generate alpha and beta dynamics. The visual cortex has been suggested to act as a dynamic filter of the visual input where stimulus properties like movement, contrast, localization and size of visual cues may modify the configuration of gamma oscillation (Gray et al., 1989; Ray and Maunsell, 2010; Brunet et al., 2013; Roberts et al., 2013). Among these stimulus properties, contrast is able to enhanced the signal-to-noise ratio of the sensory input inducing an increase in the postsynaptic gain of superficial pyramidal cells implicate in gamma oscillation (Feldman and Friston, 2010). Although, the same avatar was used here in the three different conditions the kinematic change of the *Uncoordinated* condition induced a significant increase in the dynamic contrast at the latency of 400 ms and may thus explain the late gamma ERD present in this condition. The spatial summation and the receptive field organization in V1 depend on contrast stimulus (Sceniak et al., 2002). The effects of contrast on the induced rhythms are complex and specifically influence the postsynaptic gain of neuronal populations, the strength of intrinsic and horizontal connectivity which can be differentially engaged depending on stimulus properties (Pinotsis et al., 2014). These authors have reported that the increase in visual contrast induces an increase of gamma peak frequency (from 46 to 58 Hz) accompanied by a decrease in gamma power. This contrast effect on the gamma power must be taken in account in the present gamma ERD and is complementary to the previous Kruse and Eckhorn's (1996) reported effect on the gamma power when the visual movement is *Uncoordinated*.

### SSVEP

In order to strengthen the ERP, ERSP, and ITC studies of the transient presentation of the walking video, a SSVEP approach was made by using the heel strike events as the synchronized item of the video images occurring at every 100 ms. SSVEP offer many advantages in comparison to ERP, including better signal-to-noise ratio with a clear peak in the FFT occurring at the frequency of interest and some of its harmonics, and greater number of averaged items in a shorter period of time. SSVEP are classically obtained by using neutral LED or LCD image flashing between 1 and 100 Hz inducing resonance phenomena. In the present study, the SSVEP was not obtained by directly triggering all of the images occurring at 10 Hz but by using a specific event of the avatar locomotion corresponding to the initiation of the step cycle.

SSVEP is classically considered as an oscillatory response of the visual cortex evoked by contrast or luminance-modulated stimuli that drive the neural response at the imposed frequency of the constant peripheral stimulation (Regan, 1966; Müller et al., 1998). SSVEP are not only imposed by the physical properties of the stimulus but also depends on the brain state, task related-process, bottom-up and top-down influences (Müller et al., 1998; Keil et al., 2003; Andersen and Müller, 2010). This oscillatory pattern is a strong steady state potential that mainly arises from the occipital area, with strong contribution from the early visual cortex but also from more extended parts of the visual system including higher visual areas (Müller et al., 1997; Di Russo et al., 2007). This partly explains why SSVEP approach is increasingly used in cognitive and affective neurosciences to study face processing including face identification and decoding of facial emotional expressions (McTeague et al., 2011; Ales et al., 2012; Gruss et al., 2012; Rossion et al., 2012). To our knowledge, the present study is the first to use a walking avatar video for inducing SSVEP-like response. It showed specific amplitude enhancement of the oscillatory pattern and the related spectral perturbation at a precise time in relation to a kinematic event. Although no direct comparison can be made between SSVEP and ERP results, it is interesting to highlight the convergence of both types of results with regard to significant changes in the EEG brain rhythms at about the latency of 300 ms when the same avatar video was presented in *Upside-down* vs. *Normal* condition. The reported differences in the SSVEP configuration and rhythmic alteration (early theta-alpha ERS and late gamma ERS) in the *Uncoordinated* condition can be due to the higher dynamic contrast of this condition with respect to the other two conditions.

### Conflict of interest statement

The authors declare that the research was conducted in the absence of any commercial or financial relationships that could be construed as a potential conflict of interest.

## References

[B1] Akam T. Kullmann D. M. (2014). Oscillatory multiplexing of population codes for selective communication in the mammalian brain. Nat. Rev. Neurosci. 15, 111–122 10.1038/nrn3668 24434912 PMC4724886

[B2] Ales J. M. Farzin F. Rossion B. Norcia A. M. (2012). An objective method for measuring face detection thresholds using the sweep steady-state visual evoked response. J. Vis. 12:18 10.1167/12.10.18 23024355 PMC4507785

[B3] Andersen S. K. Müller M. M. (2010). Behavioral performance follows the time course of neural facilitation and suppression during cued shifts of feature-selective attention. Proc. Natl. Acad. Sci. U.S.A. 107, 13878–13882 10.1073/pnas.1002436107 20643918 PMC2922290

[B4] Arnstein D. Cui F. Keysers C. Maurits N. M. Gazzola V. (2011). μ-suppression during action observation and execution correlates with BOLD in dorsal premotor, inferior parietal, and SI cortices. J. Neurosci. 31, 14243–14249 10.1523/JNEUROSCI.0963-11.2011 21976509 PMC6623646

[B5] Aru J. Axmacher N. Do Lam A. T. A. Fell J. Elger C. E. Singer W. (2012). Local category-specific gamma band responses in the visual cortex do not reflect conscious perception. J. Neurosci. 32, 14909–14914 10.1523/JNEUROSCI.2051-12.2012 23100413 PMC6704831

[B6] Avanzini P. Fabbri-Destro M. Campi C. Pascarella A. Barchiesi G. Cattaneo L. (2013). Spatiotemporal dynamics in understanding hand-object interactions. Proc. Natl. Acad. Sci. U.S.A. 110, 15878–15885 10.1073/pnas.1314420110 24043805 PMC3791766

[B7] Avanzini P. Fabbri-Destro M. Dalla Volta R. Daprati E. Rizzolatti G. Cantalupo G. (2012). The dynamics of sensorimotor cortical oscillations during the observation of hand movements: an EEG study. PLoS ONE 7:e37534 10.1371/journal.pone.0037534 22624046 PMC3356327

[B8] Baars B. J. (1997). Spatial brain coherence during the establishment of a conscious event. Conscious. Cogn. 6, 1–2 10.1006/ccog.1996.0289 9170557

[B9] Baars B. J. Franklin S. Ramsoy T. Z. (2013). Global workspace dynamics: cortical “binding and propagation” enables conscious contents. Front. Psychol. 4:200 10.3389/fpsyg.2013.00200 23974723 PMC3664777

[B10] Baccus W. Mozgova O. Thompson J. C. (2009). Early integration of form and motion in the neural response to biological motion. Neuroreport 20, 1334–1338 10.1097/WNR.0b013e328330a867 19687766

[B11] Bastos A. M. Usrey W. M. Adams R. A. Mangun G. R. Fries P. Friston K. J. (2012). Canonical microcircuits for predictive coding. Neuron 76, 695–711 10.1016/j.neuron.2012.10.038 23177956 PMC3777738

[B13] Blake R. Shiffrar M. (2007). Perception of human motion. Annu. Rev. Psychol. 58, 47–73 10.1146/annurev.psych.57.102904.190152 16903802

[B14] Blatow M. Rozov A. Katona I. Hormuzdi S. G. Meyer A. H. Whittington M. A. (2003). A novel network of multipolar bursting interneurons generates theta frequency oscillations in neocortex. Neuron 38, 805–817 10.1016/S0896-6273(03)00300-3 12797964

[B15] Braadbaart L. Williams J. H. G. Waiter G. D. (2013). Do mirror neuron areas mediate mu rhythm suppression during imitation and action observation? Int. J. Psychophysiol. 89, 99–105 10.1016/j.ijpsycho.2013.05.019 23756148

[B16] Brunel N. Wang X.-J. (2003). What determines the frequency of fast network oscillations with irregular neural discharges? I. Synaptic dynamics and excitation-inhibition balance. J. Neurophysiol. 90, 415–430 10.1152/jn.01095.2002 12611969

[B17] Brunet N. Bosman C. A. Roberts M. Oostenveld R. Womelsdorf T. De Weerd P. (2013). Visual cortical gamma-band activity during free viewing of natural images. Cereb. Cortex. [Epub ahead of print]. 10.1093/cercor/bht280 24108806 PMC4379996

[B18] Brunner C. Delorme A. Makeig S. (2013). Eeglab—an open source matlab toolbox for electrophysiological research. Biomed. Tech. (Berl). [Epub ahead of print]. 10.1515/bmt-2013-4182 24042816

[B19] Buzsáki G. (2002). Theta oscillations in the hippocampus. Neuron 33, 325–340 10.1016/S0896-6273(02)00586-X 11832222

[B20] Buzsáki G. Draguhn A. (2004). Neuronal oscillations in cortical networks. Science 304, 1926–1929 10.1126/science.1099745 15218136

[B21] Buzzell G. Chubb L. Safford A. S. Thompson J. C. McDonald C. G. (2013). Speed of human biological form and motion processing. PLoS ONE 8:e69396 10.1371/journal.pone.0069396 23894467 PMC3722264

[B22] Caplan J. B. Madsen J. R. Raghavachari S. Kahana M. J. (2001). Distinct patterns of brain oscillations underlie two basic parameters of human maze learning. J. Neurophysiol. 86, 368–380 11431517 10.1152/jn.2001.86.1.368

[B23] Cardin V. Friston K. J. Zeki S. (2011). Top-down modulations in the visual form pathway revealed with dynamic causal modeling. Cereb. Cortex 21, 550–562 10.1093/cercor/bhq122 20621984 PMC3041008

[B24] Cebolla A. M. De Saedeleer C. Bengoetxea A. Leurs F. Balestra C. d' Alcantara P. (2009). Movement gating of beta/gamma oscillations involved in the N30 somatosensory evoked potential. Hum. Brain Mapp. 30, 1568–1579 10.1002/hbm.20624 18661507 PMC6870656

[B25] Cebolla A. M. Palmero-Soler E. Dan B. Cheron G. (2014). Modulation of the N30 generators of the somatosensory evoked potentials by the mirror neuron system. Neuroimage 95C, 48–60 10.1016/j.neuroimage.2014.03.039 24662578

[B26] Cheng Y.-W. Tzeng O. J. L. Hung D. Decety J. Hsieh J.-C. (2005). Modulation of spinal excitability during observation of bipedal locomotion. Neuroreport 16, 1711–1714 10.1097/01.wnr.0000183325.13618.5f 16189483

[B27] Cheron G. Cebolla A. M. De Saedeleer C. Bengoetxea A. Leurs F. Leroy A. (2007). Pure phase-locking of beta/gamma oscillation contributes to the N30 frontal component of somatosensory evoked potentials. BMC Neurosci. 8:75 10.1186/1471-2202-8-75 17877800 PMC2075516

[B28] Cheron G. Leroy A. De Saedeleer C. Bengoetxea A. Lipshits M. Cebolla A. (2006). Effect of gravity on human spontaneous 10-Hz electroencephalographic oscillations during the arrest reaction. Brain Res. 1121, 104–116 10.1016/j.brainres.2006.08.098 17034767

[B29] Cheron G. Leroy A. Palmero-Soler E. De Saedeleer C. Bengoetxea A. Cebolla A.-M. (2014). Gravity influences top-down signals in visual processing. PLoS ONE 9:e82371 10.1371/journal.pone.0082371 24400069 PMC3882212

[B30] Cochin S. Barthelemy C. Lejeune B. Roux S. Martineau J. (1998). Perception of motion and qEEG activity in human adults. Electroencephalogr. Clin. Neurophysiol. 107, 287–295 10.1016/S0013-4694(98)00071-6 9872446

[B32] Crompton R. H. Sellers W. I. Thorpe S. K. S. (2010). Arboreality, terrestriality and bipedalism. Philos. Trans. R. Soc. Lond. B Biol. Sci. 365, 3301–3314 10.1098/rstb.2010.0035 20855304 PMC2981953

[B33] Csibra G. (2007). Action mirroring and action interpretation: an alternative account, in Sensorimotor Foundations of Higher Cognition. Attention and Performance XXII, eds Haggard P. Rosetti Y. Kawato M. (Oxford; New York, NY: Oxford University Press), 435–459

[B34] Darwin C. (1872). The Expression of the Emotions in Man and Animals. 1st Edn London: John Murray

[B35] De Gelder B. (2006). Towards the neurobiology of emotional body language. Nat. Rev. Neurosci. 7, 242–249 10.1038/nrn1872 16495945

[B36] Dehaene S. Charles L. King J.-R. Marti S. (2014). Toward a computational theory of conscious processing. Curr. Opin. Neurobiol. 25C, 76–84 10.1016/j.conb.2013.12.005 24709604 PMC5635963

[B37] Dehaene S. Naccache L. (2001). Towards a cognitive neuroscience of consciousness: basic evidence and a workspace framework. Cognition 79, 1–37 10.1016/S0010-0277(00)00123-2 11164022

[B38] Delorme A. Makeig S. (2004). EEGLAB: an open source toolbox for analysis of single-trial EEG dynamics including independent component analysis. J. Neurosci. Methods 134, 9–21 10.1016/j.jneumeth.2003.10.009 15102499

[B39] Di Dio C. Di Cesare G. Higuchi S. Roberts N. Vogt S. Rizzolatti G. (2013). The neural correlates of velocity processing during the observation of a biological effector in the parietal and premotor cortex. Neuroimage 64, 425–436 10.1016/j.neuroimage.2012.09.026 22995779

[B40] Di Russo F. Pitzalis S. Aprile T. Spitoni G. Patria F. Stella A. (2007). Spatiotemporal analysis of the cortical sources of the steady-state visual evoked potential. Hum. Brain Mapp. 28, 323–334 10.1002/hbm.20276 16779799 PMC6871301

[B41] Downing P. E. Jiang Y. Shuman M. Kanwisher N. (2001). A cortical area selective for visual processing of the human body. Science 293, 2470–2473 10.1126/science.1063414 11577239

[B42] Eckhorn R. Bauer R. Jordan W. Brosch M. Kruse W. Munk M. (1988). Coherent oscillations: a mechanism of feature linking in the visual cortex? Multiple electrode and correlation analyses in the cat. Biol. Cybern. 60, 121–130 10.1007/BF00202899 3228555

[B43] Ekstrom A. D. Caplan J. B. Ho E. Shattuck K. Fried I. Kahana M. J. (2005). Human hippocampal theta activity during virtual navigation. Hippocampus 15, 881–889 10.1002/hipo.20109 16114040

[B45] Engel A. K. Roelfsema P. R. Fries P. Brecht M. Singer W. (1997). Role of the temporal domain for response selection and perceptual binding. Cereb. Cortex 7, 571–582 10.1093/cercor/7.6.571 9276181

[B48] Feldman H. Friston K. J. (2010). Attention, uncertainty, and free-energy. Front. Hum. Neurosci. 4:215 10.3389/fnhum.2010.00215 21160551 PMC3001758

[B49] Flint A. C. Connors B. W. (1996). Two types of network oscillations in neocortex mediated by distinct glutamate receptor subtypes and neuronal populations. J. Neurophysiol. 75, 951–957 8714667 10.1152/jn.1996.75.2.951

[B51] Frenkel-Toledo S. Bentin S. Perry A. Liebermann D. G. Soroker N. (2014). Mirror-neuron system recruitment by action observation: effects of focal brain damage on mu suppression. Neuroimage 87, 127–137 10.1016/j.neuroimage.2013.10.019 24140938

[B52] Friedman-Hill S. Maldonado P. E. Gray C. M. (2000). Dynamics of striate cortical activity in the alert macaque: I. Incidence and stimulus-dependence of gamma-band neuronal oscillations. Cereb. Cortex 10, 1105–1116 10.1093/cercor/10.11.1105 11053231

[B53] Fries P. (2005). A mechanism for cognitive dynamics: neuronal communication through neuronal coherence. Trends Cogn. Sci. 9, 474–480 10.1016/j.tics.2005.08.011 16150631

[B54] Fries P. (2009). Neuronal gamma-band synchronization as a fundamental process in cortical computation. Annu. Rev. Neurosci. 32, 209–224 10.1146/annurev.neuro.051508.135603 19400723

[B55] Fries P. Neuenschwander S. Engel A. K. Goebel R. Singer W. (2001a). Rapid feature selective neuronal synchronization through correlated latency shifting. Nat. Neurosci. 4, 194–200 10.1038/84032 11175881

[B56] Fries P. Reynolds J. H. Rorie A. E. Desimone R. (2001b). Modulation of oscillatory neuronal synchronization by selective visual attention. Science 291, 1560–1563 10.1126/science.1055465 11222864

[B57] Fries P. Schröder J.-H. Roelfsema P. R. Singer W. Engel A. K. (2002). Oscillatory neuronal synchronization in primary visual cortex as a correlate of stimulus selection. J. Neurosci. 22, 3739–3754 11978850 10.1523/JNEUROSCI.22-09-03739.2002 PMC6758402

[B58] Fries P. Womelsdorf T. Oostenveld R. Desimone R. (2008). The effects of visual stimulation and selective visual attention on rhythmic neuronal synchronization in macaque area V4. J. Neurosci. 28, 4823–4835 10.1523/JNEUROSCI.4499-07.2008 18448659 PMC3844818

[B59] Gevins A. Smith M. E. McEvoy L. Yu D. (1997). High-resolution EEG mapping of cortical activation related to working memory: effects of task difficulty, type of processing, and practice. Cereb. Cortex 7, 374–385 10.1093/cercor/7.4.374 9177767

[B60] Giese M. A. Poggio T. (2003). Neural mechanisms for the recognition of biological movements. Nat. Rev. Neurosci. 4, 179–192 10.1038/nrn1057 12612631

[B61] Girges C. Wright M. J. Spencer J. V. O'Brien J. M. D. (2014). Event-related alpha suppression in response to facial motion. PLoS ONE 9:e89382 10.1371/journal.pone.0089382 24586735 PMC3929715

[B62] Gramann K. El Sharkawy J. Deubel H. (2009). Eye-movements during navigation in a virtual tunnel. Int. J. Neurosci. 119, 1755–1778 10.1080/00207450903170361 19922385

[B63] Gray C. M. König P. Engel A. K. Singer W. (1989). Oscillatory responses in cat visual cortex exhibit inter-columnar synchronization which reflects global stimulus properties. Nature 338, 334–337 10.1038/338334a0 2922061

[B64] Gruss L. F. Wieser M. J. Schweinberger S. Keil A. (2012). Face-evoked steady-state visual potentials: effects of presentation rate and face inversion. Front. Hum. Neurosci. 6:316 10.3389/fnhum.2012.00316 23205009 PMC3506985

[B66] Guitart-Masip M. Barnes G. R. Horner A. Bauer M. Dolan R. J. Duzel E. (2013). Synchronization of medial temporal lobe and prefrontal rhythms in human decision making. J. Neurosci. 33, 442–451 10.1523/JNEUROSCI.2573-12.2013 23303925 PMC3562870

[B67] Haegens S. Osipova D. Oostenveld R. Jensen O. (2010). Somatosensory working memory performance in humans depends on both engagement and disengagement of regions in a distributed network. Hum. Brain Mapp. 31, 26–35 10.1002/hbm.20842 19569072 PMC6871021

[B68] Heyes C. (2010). Where do mirror neurons come from? Neurosci. Biobehav. Rev. 34, 575–583 10.1016/j.neubiorev.2009.11.007 19914284

[B69] Hirai M. Fukushima H. Hiraki K. (2003). An event-related potentials study of biological motion perception in humans. Neurosci. Lett. 344, 41–44 10.1016/S0304-3940(03)00413-0 12781917

[B70] Hirai M. Hiraki K. (2006). The relative importance of spatial versus temporal structure in the perception of biological motion: an event-related potential study. Cognition 99, B15–B29 10.1016/j.cognition.2005.05.003 16051211

[B71] Hirai M. Senju A. Fukushima H. Hiraki K. (2005). Active processing of biological motion perception: an ERP study. Brain Res. Cogn. Brain Res. 23, 387–396 10.1016/j.cogbrainres.2004.11.005 15820645

[B72] Hirai M. Watanabe S. Honda Y. Kakigi R. (2009). Developmental changes in point-light walker processing during childhood and adolescence: an event-related potential study. Neuroscience 161, 311–325 10.1016/j.neuroscience.2009.03.026 19303916

[B73] Hirai M. Watanabe S. Honda Y. Kakigi R. (2013). Developmental changes in point-light walker processing during childhood: a two-year follow-up ERP study. Dev. Cogn. Neurosci. 5, 51–62 10.1016/j.dcn.2013.01.002 23376474 PMC6987752

[B74] Howard M. W. Rizzuto D. S. Caplan J. B. Madsen J. R. Lisman J. Aschenbrenner-Scheibe R. (2003). Gamma oscillations correlate with working memory load in humans. Cereb. Cortex 13, 1369–1374 10.1093/cercor/bhg084 14615302

[B75] Ishihara T. Yoshi N. (1972). Multivariate analytic study of EEG and mental activity in juvenile delinquents. Electroencephalogr. Clin. Neurophysiol. 33, 71–80 10.1016/0013-4694(72)90026-0 4113276

[B76] Jacob P. Jeannerod M. (2005). The motor theory of social cognition: a critique. Trends Cogn. Sci. 9, 21–25 10.1016/j.tics.2004.11.003 15639437

[B77] Jeffreys D. A. (1996). Simple methods of identifying the independently generated components of scalp-recorded responses evoked by stationary patterns. Exp. Brain Res. 111, 100–112 10.1007/BF00229559 8891640

[B78] Jensen O. Colgin L. L. (2007). Cross-frequency coupling between neuronal oscillations. Trends Cogn. Sci. 11, 267–269 10.1016/j.tics.2007.05.003 17548233

[B79] Johansson G. (1973). Visual perception of biological motion and a model for its analysis. Percept. Psychophys. 14, 201–211 10.3758/BF03212378

[B80] Jokisch D. Daum I. Suchan B. Troje N. F. (2005). Structural encoding and recognition of biological motion: evidence from event-related potentials and source analysis. Behav. Brain Res. 157, 195–204 10.1016/j.bbr.2004.06.025 15639170

[B81] Kahana M. J. Sekuler R. Caplan J. B. Kirschen M. Madsen J. R. (1999). Human theta oscillations exhibit task dependence during virtual maze navigation. Nature 399, 781–784 10.1038/21645 10391243

[B82] Kandel E. (2013). The new science of mind and the future of knowledge. Neuron 80, 546–560 10.1016/j.neuron.2013.10.039 24183008

[B83] Kang J. Robinson H. P. C. Feng J. (2010). Diversity of intrinsic frequency encoding patterns in rat cortical neurons—mechanisms and possible functions. PLoS ONE 5:e9608 10.1371/journal.pone.0009608 20333256 PMC2841633

[B84] Keil A. Gruber T. Müller M. M. Moratti S. Stolarova M. Bradley M. M. (2003). Early modulation of visual perception by emotional arousal: evidence from steady state visual evoked brain potentials. Cogn. Affect. Behav. Neurosci. 3, 195–206 10.3758/CABN.3.3.195 14672156

[B85] Kilner J. M. Friston K. J. Frith C. D. (2007). Predictive coding: an account of the mirror neuron system. Cogn. Process. 8, 159–166 10.1007/s10339-007-0170-2 17429704 PMC2649419

[B86] Klimesch W. (1999). EEG alpha and theta oscillations reflect cognitive and memory performance: a review and analysis. Brain Res. Brain Res. Rev. 29, 169–195 10.1016/S0165-0173(98)00056-3 10209231

[B87] Klimesch W. (2012). Alpha-band oscillations, attention, and controlled access to stored information. Trends Cogn. Sci. 16, 606–617 10.1016/j.tics.2012.10.007 23141428 PMC3507158

[B90a] Klimesch W. Doppelmayr M. Pachinger T. Russegger H. (1997). Event-related desynchronization in the alpha band and the processing of semantic information. Brain Res. Cogn. Brain Res. 6, 83–94 10.1016/S0926-6410(97)00018-9 9450602

[B88] Klimesch W. Doppelmayr M. Schimke H. Pachinger T. (1996). Alpha frequency, reaction time, and the speed of processing information. J. Clin. Neurophysiol. 13, 511–518 8978623 10.1097/00004691-199611000-00006

[B89] Klimesch W. Sauseng P. Gerloff C. (2003). Enhancing cognitive performance with repetitive transcranial magnetic stimulation at human individual alpha frequency. Eur. J. Neurosci. 17, 1129–1133 10.1046/j.1460-9568.2003.02517.x 12653991

[B90] Klimesch W. Sauseng P. Hanslmayr S. (2007). EEG alpha oscillations: the inhibition-timing hypothesis. Brain Res. Rev. 53, 63–88 10.1016/j.brainresrev.2006.06.003 16887192

[B91] Klimesch W. Schack B. Schabus M. Doppelmayr M. Gruber W. Sauseng P. (2004). Phase-locked alpha and theta oscillations generate the P1-N1 complex and are related to memory performance. Brain Res. Cogn. Brain Res. 19, 302–316 10.1016/j.cogbrainres.2003.11.016 15062867

[B92] Kocsis B. Bragin A. Buzsáki G. (1999). Interdependence of multiple theta generators in the hippocampus: a partial coherence analysis. J. Neurosci. 19, 6200–6212 10407056 10.1523/JNEUROSCI.19-14-06200.1999 PMC6783086

[B93] Krakowski A. I. Ross L. A. Snyder A. C. Sehatpour P. Kelly S. P. Foxe J. J. (2011). The neurophysiology of human biological motion processing: a high-density electrical mapping study. Neuroimage 56, 373–383 10.1016/j.neuroimage.2011.01.058 21276862 PMC6589837

[B94] Kravitz D. J. Saleem K. S. Baker C. I. Mishkin M. (2011). A new neural framework for visuospatial processing. Nat. Rev. Neurosci. 12, 217–230 10.1038/nrn3008 21415848 PMC3388718

[B95] Kruse W. Eckhorn R. (1996). Inhibition of sustained gamma oscillations (35-80 Hz) by fast transient responses in cat visual cortex. Proc. Natl. Acad. Sci. U.S.A. 93, 6112–6117 10.1073/pnas.93.12.6112 8650228 PMC39198

[B96] Leakey M. Walker A. (1997). Early hominid fossils from Africa. Sci. Am. 276, 74–79 10.1038/scientificamerican0697-74 9198897

[B97] Leung L. W. Borst J. G. (1987). Electrical activity of the cingulate cortex. I. Generating mechanisms and relations to behavior. Brain Res. 407, 68–80 10.1016/0006-8993(87)91220-0 3580857

[B98] Liebe S. Hoerzer G. M. Logothetis N. K. Rainer G. (2012). Theta coupling between V4 and prefrontal cortex predicts visual short-term memory performance. Nat. Neurosci. 15, 456–462, S1–S2. 10.1038/nn.3038 22286175

[B99] Makeig S. Westerfield M. Jung T. P. Enghoff S. Townsend J. Courchesne E. (2002). Dynamic brain sources of visual evoked responses. Science 295, 690–694 10.1126/science.1066168 11809976

[B100] McAleer P. Pollick F. E. Love S. A. Crabbe F. Zacks J. M. (2014). The role of kinematics in cortical regions for continuous human motion perception. Cogn. Affect. Behav. Neurosci. 14, 307–318 10.3758/s13415-013-0192-4 23943513 PMC8679008

[B101] McGlothlin B. Jiacoletti D. Yandell L. (2012). The inversion effect: biological motion and gender recognition. Psi Chi J. Psychol. Res. 17, 68–72

[B102] McTeague L. M. Shumen J. R. Wieser M. J. Lang P. J. Keil A. (2011). Social vision: sustained perceptual enhancement of affective facial cues in social anxiety. Neuroimage 54, 1615–1624 10.1016/j.neuroimage.2010.08.080 20832490 PMC3004773

[B103] Mitchell D. J. McNaughton N. Flanagan D. Kirk I. J. (2008). Frontal-midline theta from the perspective of hippocampal “theta.” Prog. Neurobiol. 86, 156–185 10.1016/j.pneurobio.2008.09.005 18824212

[B104] Mizuhara H. Wang L.-Q. Kobayashi K. Yamaguchi Y. (2004). A long-range cortical network emerging with theta oscillation in a mental task. Neuroreport 15, 1233–1238 10.1097/01.wnr.0000126755.09715.b3 15167540

[B105] Müller M. M. Teder-Salejarvi W. Hillyard S. A. (1998). The time course of cortical facilitation during cued shifts of spatial attention. Nat. Neurosci. 1, 631–634 10.1038/2865 10196572

[B106] Müller M. M. Teder W. Hillyard S. A. (1997). Magnetoencephalographic recording of steady-state visual evoked cortical activity. Brain Topogr. 9, 163–168 10.1007/BF01190385 9104827

[B107] O'Keefe J. Recce M. L. (1993). Phase relationship between hippocampal place units and the EEG theta rhythm. Hippocampus 3, 317–330 8353611 10.1002/hipo.450030307

[B108] Pavlova M. Birbaumer N. Sokolov A. (2006). Attentional modulation of cortical neuromagnetic gamma response to biological movement. Cereb. Cortex 16, 321–327 10.1093/cercor/bhi108 15901655

[B109] Pavlova M. Lutzenberger W. Sokolov A. Birbaumer N. (2004). Dissociable cortical processing of recognizable and non-recognizable biological movement: analysing gamma MEG activity. Cereb. Cortex 14, 181–188 10.1093/cercor/bhg117 14704215

[B110] Pavlova M. Sokolov A. (2000). Orientation specificity in biological motion perception. Percept. Psychophys. 62, 889–899 10.3758/BF03212075 10997036

[B111] Peyrin C. Michel C. M. Schwartz S. Thut G. Seghier M. Landis T. (2010). The neural substrates and timing of top-down processes during coarse-to-fine categorization of visual scenes: a combined fMRI and ERP study. J. Cogn. Neurosci. 22, 2768–2780 10.1162/jocn.2010.21424 20044901

[B112] Pinotsis D. A. Brunet N. Bastos A. Bosman C. A. Litvak V. Fries P. (2014). Contrast gain control and horizontal interactions in V1: a DCM study. Neuroimage 92C, 143–155 10.1016/j.neuroimage.2014.01.047 24495812 PMC4010674

[B113] Pollick F. E. Kay J. W. Heim K. Stringer R. (2005). Gender recognition from point-light walkers. J. Exp. Psychol. Hum. Percept. Perform. 31, 1247–1265 10.1037/0096-1523.31.6.1247 16366787

[B114] Pozzo T. Berthoz A. Lefort L. (1990). Head stabilization during various locomotor tasks in humans. I. Normal subjects. Exp. Brain Res. 82, 97–106 10.1007/BF00230842 2257917

[B115] Press C. Heyes C. Kilner J. M. (2011). Learning to understand others' actions. Biol. Lett. 7, 457–460 10.1098/rsbl.2010.0850 21084333 PMC3097844

[B116] Raghavachari S. Kahana M. J. Rizzuto D. S. Caplan J. B. Kirschen M. P. Bourgeois B. (2001). Gating of human theta oscillations by a working memory task. J. Neurosci. 21, 3175–3183 11312302 10.1523/JNEUROSCI.21-09-03175.2001 PMC6762557

[B117] Raghavachari S. Lisman J. E. Tully M. Madsen J. R. Bromfield E. B. Kahana M. J. (2006). Theta oscillations in human cortex during a working-memory task: evidence for local generators. J. Neurophysiol. 95, 1630–1638 10.1152/jn.00409.2005 16207788

[B118] Ramalingam N. McManus J. N. J. Li W. Gilbert C. D. (2013). Top-down modulation of lateral interactions in visual cortex. J. Neurosci. 33, 1773–1789 10.1523/JNEUROSCI.3825-12.2013 23365217 PMC3711382

[B119] Ray S. Maunsell J. H. R. (2010). Differences in gamma frequencies across visual cortex restrict their possible use in computation. Neuron 67, 885–896 10.1016/j.neuron.2010.08.004 20826318 PMC3001273

[B120] Regan D. (1966). Some characteristics of average steady-state and transient responses evoked by modulated light. Electroencephalogr. Clin. Neurophysiol. 20, 238–248 10.1016/0013-4694(66)90088-5 4160391

[B121] Rey H. G. Fried I. Quian Quiroga R. (2014). Timing of single-neuron and local field potential responses in the human medial temporal lobe. Curr. Biol. 24, 299–304 10.1016/j.cub.2013.12.004 24462002 PMC3963414

[B122] Rizzolatti G. Craighero L. (2004). The mirror-neuron system. Annu. Rev. Neurosci. 27, 169–192 10.1146/annurev.neuro.27.070203.144230 15217330

[B123] Rizzolatti G. Fadiga L. Gallese V. Fogassi L. (1996). Premotor cortex and the recognition of motor actions. Brain Res. Cogn. Brain Res. 3, 131–141 10.1016/0926-6410(95)00038-0 8713554

[B124] Roberts M. J. Lowet E. Brunet N. M. Ter Wal M. Tiesinga P. Fries P. (2013). Robust gamma coherence between macaque V1 and V2 by dynamic frequency matching. Neuron 78, 523–536 10.1016/j.neuron.2013.03.003 23664617

[B125] Rojas M. Masip D. Todorov A. Vitria J. (2011). Automatic prediction of facial trait judgments: appearance vs. structural models. PLoS ONE 6:e23323 10.1371/journal.pone.0023323 21858069 PMC3157350

[B126] Rossion B. Prieto E. A. Boremanse A. Kuefner D. Van Belle G. (2012). A steady-state visual evoked potential approach to individual face perception: effect of inversion, contrast-reversal and temporal dynamics. Neuroimage 63, 1585–1600 10.1016/j.neuroimage.2012.08.033 22917988

[B127] Sadaghiani S. Scheeringa R. Lehongre K. Morillon B. Giraud A.-L. D'Esposito M. (2012). α-band phase synchrony is related to activity in the fronto-parietal adaptive control network. J. Neurosci. 32, 14305–14310 10.1523/JNEUROSCI.1358-12.2012 23055501 PMC4057938

[B128] Sasaki K. Gemba H. Nambu A. Kyuhou S. Matsuzaki R. Tsujimoto T. (1996a). Studies on integrative functions of the human frontal association cortex by use of MEG. Electroencephalogr. Clin. Neurophysiol. Suppl. 47, 181–190 9335982

[B129] Sasaki K. Nambu A. Tsujimoto T. Matsuzaki R. Kyuhou S. Gemba H. (1996b). Studies on integrative functions of the human frontal association cortex with MEG. Brain Res. Cogn. Brain Res. 5, 165–174 10.1016/S0926-6410(96)00053-5 9049083

[B130] Sasaki K. Tsujimoto T. Nishikawa S. Nishitani N. Ishihara T. (1996c). Frontal mental theta wave recorded simultaneously with magnetoencephalography and electroencephalography. Neurosci. Res. 26, 79–81 10.1016/0168-0102(96)01082-6 8895895

[B131] Sauseng P. Hoppe J. Klimesch W. Gerloff C. Hummel F. C. (2007). Dissociation of sustained attention from central executive functions: local activity and interregional connectivity in the theta range. Eur. J. Neurosci. 25, 587–593 10.1111/j.1460-9568.2006.05286.x 17284201

[B132] Saygin A. P. Wilson S. M. Hagler D. J. Jr. Bates E. Sereno M. I. (2004). Point-light biological motion perception activates human premotor cortex. J. Neurosci. 24, 6181–6188 10.1523/JNEUROSCI.0504-04.2004 15240810 PMC6729669

[B133] Sceniak M. P. Hawken M. J. Shapley R. (2002). Contrast-dependent changes in spatial frequency tuning of macaque V1 neurons: effects of a changing receptive field size. J. Neurophysiol. 88, 1363–1373 12205157 10.1152/jn.2002.88.3.1363

[B134] Schütz-Bosbach S. Prinz W. (2007). Perceptual resonance: action-induced modulation of perception. Trends Cogn. Sci. 11, 349–355 10.1016/j.tics.2007.06.005 17629544

[B135] Sederberg P. B. Kahana M. J. Howard M. W. Donner E. J. Madsen J. R. (2003). Theta and gamma oscillations during encoding predict subsequent recall. J. Neurosci. 23, 10809–10814 14645473 10.1523/JNEUROSCI.23-34-10809.2003 PMC6740970

[B136] Silva L. R. Amitai Y. Connors B. W. (1991). Intrinsic oscillations of neocortex generated by layer 5 pyramidal neurons. Science 251, 432–435 10.1126/science.1824881 1824881

[B137] Singer W. (1999). Neuronal synchrony: a versatile code for the definition of relations? Neuron 24, 49–65, 111–125. 10.1016/S0896-6273(00)80821-1 10677026

[B138] Singer W. (2009). Distributed processing and temporal codes in neuronal networks. Cogn. Neurodyn. 3, 189–196 10.1007/s11571-009-9087-z 19562517 PMC2727167

[B140] Tallon-Baudry C. Bertrand O. (1999). Oscillatory gamma activity in humans and its role in object representation. Trends Cogn. Sci. 3, 151–162 10.1016/S1364-6613(99)01299-1 10322469

[B141] Tallon-Baudry C. Bertrand O. Delpuech C. Pernier J. (1996). Stimulus specificity of phase-locked and non-phase-locked 40 Hz visual responses in human. J. Neurosci. 16, 4240–4249 8753885 10.1523/JNEUROSCI.16-13-04240.1996 PMC6579008

[B142] Thorpe S. K. S. Holder R. L. Crompton R. H. (2007). Origin of human bipedalism as an adaptation for locomotion on flexible branches. Science 316, 1328–1331 10.1126/science.1140799 17540902

[B143] Traub R. D. Cunningham M. O. Gloveli T. LeBeau F. E. N. Bibbig A. Buhl E. H. (2003). GABA-enhanced collective behavior in neuronal axons underlies persistent gamma-frequency oscillations. Proc. Natl. Acad. Sci. U.S.A. 100, 11047–11052 10.1073/pnas.1934854100 12960382 PMC196924

[B144] Traub R. D. Jefferys J. G. Whittington M. A. (1997). Simulation of gamma rhythms in networks of interneurons and pyramidal cells. J. Comput. Neurosci. 4, 141–150 10.1023/A:1008839312043 9154520

[B145] Troje N. F. Westhoff C. Lavrov M. (2005). Person identification from biological motion: effects of structural and kinematic cues. Percept. Psychophys. 67, 667–675 10.3758/BF03193523 16134460

[B146] Ulloa E. R. Pineda J. A. (2007). Recognition of point-light biological motion: mu rhythms and mirror neuron activity. Behav. Brain Res. 183, 188–194 10.1016/j.bbr.2007.06.007 17658625

[B147] Urgen B. A. Plank M. Ishiguro H. Poizner H. Saygin A. P. (2013). EEG theta and Mu oscillations during perception of human and robot actions. Front. Neurorobotics 7:19 10.3389/fnbot.2013.00019 24348375 PMC3826547

[B148] Vaina L. M. Solomon J. Chowdhury S. Sinha P. Belliveau J. W. (2001). Functional neuroanatomy of biological motion perception in humans. Proc. Natl. Acad. Sci. U.S.A. 98, 11656–11661 10.1073/pnas.191374198 11553776 PMC58785

[B149] Vidal J. R. Perrone-Bertolotti M. Levy J. De Palma L. Minotti L. Kahane P. (2014). Neural repetition suppression in ventral occipito-temporal cortex occurs during conscious and unconscious processing of frequent stimuli. Neuroimage 95C, 129–135 10.1016/j.neuroimage.2014.03.049 24667455

[B150] Viviani P. Stucchi N. (1992). Biological movements look uniform: evidence of motor-perceptual interactions. J. Exp. Psychol. Hum. Percept. Perform. 18, 603–623 10.1037/0096-1523.18.3.603 1500865

[B151] Walter W. G. Dovey V. J. (1944). Electro-encephalography in cases of sub-cortical tumour. J. Neurol. Neurosurg. Psychiatry 7, 57–65 10.1136/jnnp.7.3-4.57 21610865 PMC1061348

[B152] White N. C. Fawcett J. M. Newman A. J. (2014). Electrophysiological markers of biological motion and human form recognition. Neuroimage 84, 854–867 10.1016/j.neuroimage.2013.09.026 24064067

[B153] Whittington M. A. Traub R. D. (2003). Interneuron diversity series: inhibitory interneurons and network oscillations *in vitro*. Trends Neurosci. 26, 676–682 10.1016/j.tins.2003.09.016 14624852

[B154] Zanto T. P. Rubens M. T. Thangavel A. Gazzaley A. (2011). Causal role of the prefrontal cortex in top-down modulation of visual processing and working memory. Nat. Neurosci. 14, 656–661 10.1038/nn.2773 21441920 PMC3083493

[B158] Zeki S. Watson J. D. Lueck C. J. Friston K. J. Kennard C. Frackowiak R. S. (1991). A direct demonstration of functional specialization in human visual cortex. J. Neurosci. 11, 641–649 2002358 10.1523/JNEUROSCI.11-03-00641.1991 PMC6575357

